# Establishment and assessment of mortality risk prediction model in patients with sepsis based on early-stage peripheral lymphocyte subsets

**DOI:** 10.18632/aging.205772

**Published:** 2024-04-25

**Authors:** Fuzhu Li, Hongtao Qu, Yimin Li, Jie Liu, Hongyun Fu

**Affiliations:** 1 The First Affiliated Hospital, Department of Neurosurgical Intensive Care Unit, Hengyang Medical School, University of South China, Hengyang, Hunan 421000, China; 2 Department of Emergency, Shenzhen United Family Hospital, Shenzhen, Guangdong 518048, China; 3 The Affiliated Nanhua Hospital, Department of Docimasiology, Hengyang Medical School, University of South China, Hengyang, Hunan 421002, China

**Keywords:** endotoxin shock/sepsis, innate lymphoid cells, CD4+ T cells, CD8+ T cells

## Abstract

This study is aimed to explore the value of lymphocyte subsets in evaluating the severity and prognosis of sepsis. The counts of lymphocytes, CD3+ T cells, CD4+ T cells, CD8+ T cells, CD19+ B cells, and NK cells significantly decreased between day 1 and day 3 in both the survivor and the non-survivor groups. The peripheral lymphocyte subsets (PLS) at day 1 were not significantly different between the survivor and the non-survivor groups. However, at day 3, the counts of lymphocytes, CD3+ T cells, CD4+ T cells, and NK cells were remarkably lower in the non-survivor group. No significant differences in CD8+ T cells, or CD19+ B cells were observed. The PLS index was independently and significantly associated with the 28-day mortality risk in septic patients (OR: 3.08, 95% CI: 1.18-9.67). Based on these clinical parameters and the PLS index, we developed a nomograph for evaluating the individual mortality risk in sepsis. The area under the curve of prediction with the PLS index was significantly higher than that from the model with only clinical parameters (0.912 vs. 0.817). Our study suggests that the decline of PLS occurred in the early stage of sepsis. The new novel PLS index can be an independent predictor of 28-day mortality in septic patients. The prediction model based on clinical parameters and the PLS index has relatively high predicting ability.

## INTRODUCTION

Sepsis, known as systemic infection, is a systemic inflammatory response syndrome with infection or highly suspected infection [1]. It is one of the common serious complications of trauma, burn, infection, and perioperative period of major surgical operations [2]. The disease progresses rapidly and is likely to turn into septic shock and multi-organ dysfunction syndrome (MODS). In recent years, continuous in-depth research on sepsis, and great progress in anti-infection treatment and organ function support technology have been achieved. Nevertheless, the high case fatality rate of sepsis (up to 30%-60%), the high treatment cost, and the large consumption of medical resources seriously affect the life quality of patients and pose a great threat to human health [3]. Therefore, it is urgent to establish a simple and efficient risk management strategy for septic patients.

Although the exact pathogenesis of sepsis is not yet understood, growing research holds that immunosuppression is the central link in the pathogenesis of sepsis [4]. It was once believed that early sepsis-induced death was a multi-organ failure caused by immune damage due to excessive inflammatory response, and early death can occur within a few hours to a few days, or even shorter time [5]. Later death of sepsis is caused by organ failure due to compensatory anti-inflammatory response-mediated immune damage, or by secondary severe infection due to severe immunosuppression, which both can occur days to weeks later [6]. However, recent genetic analyses of tissue samples from septic patients reveal that innate immune system dysfunction and acquired immune system immunosuppression during sepsis can cause simultaneous imbalance and persistence of inflammatory and anti-inflammatory responses, resulting in persistent and/or repeated infections and lasting damage to organ functions and finally in death [7, 8]. Exploring the early-stage changes of immune status can help in evaluating the prognosis of septic patients and carrying out risk management. Immune dysfunction in sepsis is mainly manifested by lymphocyte subset imbalance and dysfunction. Specifically, NK cells, B cells and T cells can all show apoptosis to varying degrees, and the count of T lymphocytes significantly decreases, including changes in the ratios of CD4+, CD8+ and CD4+/CD8+ [9, 10]. Studies show that T lymphocyte subsets are important indicators for evaluating immune function. Sepsis is a clinical syndrome with heterogeneity, high morbidity, and high mortality [11]. Timely evaluation of immune status and severity of sepsis is the prerequisite for developing individualized strategies and reducing mortality. Therefore, this study is aimed to explore the value of lymphocyte subsets in evaluating the severity and prognosis of sepsis, establish a 28-day mortality risk prediction model based on early-stage peripheral lymphocyte subsets (PLS) and provide significant reference for guiding clinical practice.

## MATERIALS AND METHODS

### Study population

A retrospective study design was utilized to collect information on septic patients admitted to the First Affiliated Hospital, Hengyang Medical School, between June 2021 and July 2023. We selected the septic patients below. Criteria for inclusion were: age >16 years old; diagnosis with sepsis according to the two indicators of Sepsis 3.0 [12]. The first indicator is the presence of suspected infection, which is determined based on whether the patient has undergone blood culture examination and received antibiotic treatment. The second indicator is the Sequential Organ Failure Assessment (SOFA) score within 24 hours of admission to the intensive care unit (ICU) [13]. Based on these two indicators, diagnosis of sepsis was made. Six types of patients were excluded: (1) readmitted patients, so only those admitted to the ICU for the first time were included; (2) undergoing cardiac surgery; (3) diagnosed with sepsis either after spending more than 24 hours in the ICU or diagnosed outside the ICU; (4) concurrent metastatic cancer; (5) ICU stay less than 24 hours; (6) missing more than 20% of the laboratory test results. This study was approved by the Institutional Research Ethics Committee of Hengyang Medical School, University of South China (202103145). Written informed consent was obtained from all the patients.

### Data collection

The following data were collected: (1) general characteristics: age, gender, body mass index (BMI = weight (kg)/height (m^2^)) [14]; (2) diagnosis at admission: pulmonary, cardiovascular disease, infectious disease, polytrauma, and gastrointestinal bleeding; (3) laboratory examination: venous blood collected within 24 hours of admission; (4) comorbidities: hypertension, diabetic mellitus, infection, chronic renal failure, and respiratory disease. The white blood cell (WBC), red blood cell (RBC), hemoglobin (Hb), red cell distribution width (RDW), platelet (Plt), neutrophil, fasting blood glucose, blood urea nitrogen, creatinine, uric acid, total bilirubin, direct bilirubin, albumin, lactate, serum sodium, serum potassium, and serum phosphorus (P) were detected using an automated biochemical analyzer. The SOFA scores at day 1, 3 and 7 were also collected. The primary follow-up outcomes were 28-day mortality and organ dysfunction, including kidney, liver, heart, respiratory, and septic shock.

### Peripheral lymphocyte subsets (PLS)

We collected 3 ml of fasting venous blood from each patient using an EDTA-K2 anticoagulant at 1^st^ and 3^rd^ day after admission. A Mindray BriCyteE6 flow cytometer and the reagents provided by Shenzhen Mindray Company were used: four-color flow cytometry reagents CD3-FITC/CD8-PE/CD45-PerCP/CD4-APC, CD3-FITC/CD16+56-PE/CD45-PerCP/CD19-APC, flow-count standard fluorescence microspheres, and an FACS lysing solution (10×). Into 2 test tubes labeled as A and B, 20 μl of CD3-FITC/CD8-PE/CD45-PerCP/CD4-APC antibodies was added to tube A, and 20 μl of CD3-FITC/CD16+56-PE/CD45-PerCP/CD19-APC antibodies was added to tube B. Then 50 μl of EDTA anticoagulated venous blood was added to the bottom of each tube. The tubes were gently vortex-treated on a vortex mixer, and incubated at room temperature in the dark for 15 minutes. Then 1× lysing solution (450 μl) was put to each tube, which was gently vortex-treated again on the vortex mixer, and incubated at room temperature in the dark for 15 minutes. After sufficient red blood cell lysis, the tubes were vortex-treated and detected on the respective machine.

### Statistical analysis

We used a standard excel sheet to collect data. Data conforming to normal distribution were represented by mean ± standard deviation, and compared between groups via *t*-test. Data not conforming to normal distribution were expressed as quartiles, and compared between groups with non-parametric test (Wilcoxon test). Counting data were expressed as percentages, and compared between groups with Chi-square test. We established a PLS index as follows [15]: the regression coefficient (β) was obtained from multivariate logistic regression, which only included PLS (lymphocytes, CD3, CD4, CD8, CD19, and NK cells). The PLS index was calculated as β1 × lymphocytes+β2 × CD3+ β3 × CD4+β4 × CD5+β5 × CD8+β1 × CD19+β6 × NK. Two 28-day mortality prediction models were built using logistic regression with calculated odds ratio (OR) and 95% confidence interval (CI). Model 1 only included clinical parameters. Model 2 included both clinical parameters and the PLS index. A nomograph was plotted to predict the individual risk probability. We drew a calibration plot to evaluate the association between the predicted value and the actual value. The receiver’s operating characteristic curve (ROC) was used to evaluate the predicting ability of the models, and area under the curve (AUC) was calculated. The decision curve analysis was used to evaluate cost-benefits from the examination of PLS.

## RESULTS

### General characteristics of non-survivor and survivor groups

**Figure 1:**
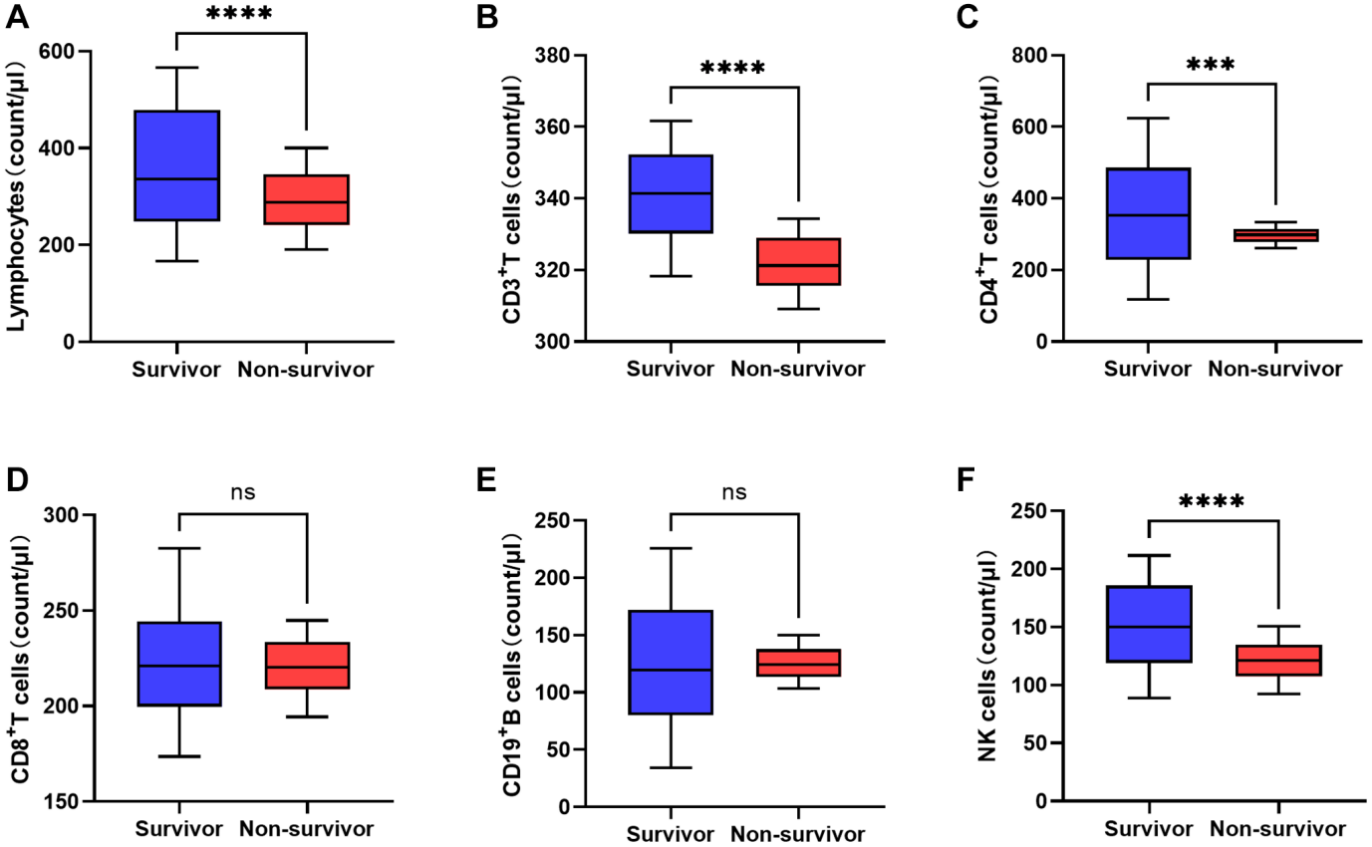
Comparison of peripheral lymphocyte subsets between non-survivor and survivor groups on the third day after admission (^*^P < 0.05, ^**^P < 0.01, ^***^P < 0.001; ^****^P < 0.0001). (**A**) Lymphocytes. (**B**) CD3+T cells. (**C**) CD4+ T cells. (**D**) CD8+ T cells. (**E**) CD19+ B cells. (**F**) NK cells.

According to the inclusion and exclusion criteria, 456 septic patients were included in the final analyses. Figure 1 presented the process of patient selection. There were 114 non-survivor cases after 28-day follow-up. The 28-day mortality rate was 25.0%. The general characteristics and laboratory examination results were presented in Table 1. There were no significant differences in age (*P* = 0.800), gender rate (*P* = 0.132), and BMI (*P* = 0.247) between the two groups. The distributions of diagnosis at admission in the survivor and the non-survivor groups were not significantly different in pulmonary disease (47.1% vs. 48.2%, *P* = 0.829), cardiovascular diseases (26.9% vs. 34.2%, *P* = 0.135), infectious disease (25.4% vs. 20.2%, *P* = 0.255), polytrauma (9.9% vs. 14.9%, *P* = 0.145), no gastrointestinal bleeding (7.0% vs. 7.9%, *P* = 0.754).

**Table 1:** Clinical characteristics of survivor and non-survivor groups.

**Parameters**	**Survivor group**	**Non-survivor group**	***P***
Age	68.3 ± 18.8	68.8 ± 20.2	0.800
Gender (male, %)	207 (60.5%)	78 (68.4%)	0.132
Body mass index (kg/m^2^)	22.4 ± 6.0	23.2 ± 8.0	0.247
**Diagnosis at admission**
Pulmonary (*n*, %)	161 (47.1%)	55 (48.2%)	0.829
Cardiovascular (*n*, %)	92 (26.9%)	39 (34.2%)	0.135
Infectious diseases (*n*, %)	87 (25.4%)	23 (20.2%)	0.255
Polytrauma (*n*, %)	34 (9.9%)	17 (14.9%)	0.145
Gastrointestinal bleeding (*n*, %)	24 (7.0%)	9 (7.9%)	0.754
**Laboratory examination (IQR)**
White blood cells (10^9^/L)	13.0 ± 3.0	12.5 ± 3.0	0.102
Red blood cells (10^9^/L)	5.9 ± 1.4	5.6 ± 1.3	0.060
Hemoglobin (g/dL)	10.4 ± 0.6	10.2 ± 0.8	**0.021**
Red cell distribution width (%)	16.6 ± 0.9	17.2 ± 1.0	**<0.001**
Platelets (10^9^/L)	133.1 ± 30.6	120.4 ± 25.3	**0.016**
Neutrophil (%)	85.3 ± 4.3	87.2 ± 8.2	**0.002**
Blood glucose (mmol/L)	138.3 ± 21.0	145.4 ± 17.2	**0.001**
Blood urea nitrogen (mg/dL)	28.7 ± 8.1	29.8 ± 5.6	0.179
Creatinine (mg/dL)	130.1 ± 37.6	139.0 ± 34.3	**0.026**
Uric acid (mg/dL)	365.2 ± 66.6	375.8 ± 87.9	0.176
Total bilirubin (mg/dL)	0.95 ± 0.3	1.05 ± 0.3	**0.006**
Direct bilirubin (mg/dL)	1.45 ± 0.5	1.56 ± 0.6	0.086
Albumin (g/dL)	2.9 ± 0.3	3.0 ± 0.4	**0.021**
Lactate (mmol/L)	2.3 ± 0.4	2.4 ± 0.4	**0.008**
Serum sodium (mmol/L)	138.3 ± 3.7	138.7 ± 4.4	0.402
Serum potassium (mmol/L)	4.1 ± 0.4	4.1 ± 0.3	0.100
Serum phosphorus (mmol/L)	3.4 ± 0.5	3.7 ± 0.5	**<0.001**
**Risk score**
Sequential organ failure assessment	7.6 ± 1.8	7.6 ± 1.9	0.920
SOFA at day 3	12.5 ± 1.4	13.0 ± 1.8	**0.001**
SOFA at day 7	14.1 ± 1.1	15.0 ± 3.1	**<0.001**
ICU stay time (days)	12.3 (6.0−18.9)	11.4 ± (6.2−18.1)	0.082
**Comorbidities**
Hypertension (%)	220 (64.3%)	78 (68.4%)	0.426
Diabetic mellitus (%)	187 (54.7%)	72 (63.2%)	0.113
Infection (%)	57 (16.7%)	24 (21.1%)	0.289
Chronic renal failure (%)	73 (21.3%)	25 (21.9%)	0.895
Respiratory disease (%)	206 (60.2%)	72 (63.2%)	0.579
**Organ dysfunction**
Kidney (%)	155 (45.3%)	62 (54.4%)	0.093
Liver (%)	63 (18.4%)	27 (23.7%)	0.221
Heart (%)	187 (54.7%)	66 (57.9%)	0.550
Respiratory (%)	350 (89.2%)	103 (90.4%)	0.725
Septic shock (*n*, %)	74 (21.6%)	65 (57.0%)	**<0.001**

The non-survivor group had lower levels of Hb (*P* = 0.021), RDW (*P* < 0.001), and Plt (*P* = 0.016) than in the survivor group. On the contrary, the levels of neutrophil (*P* = 0.002), blood glucose (*P* = 0.001), creatinine (*P* = 0.026), total bilirubin (*P* = 0.006), albumin (*P* = 0.021), lactate (*P* = 0.008), and serum phosphorus (*P* < 0.001) were significantly elevated in the non-survivor group. No significance differences were observed in WBC (*P* = 0.102), RBC (*P* = 0.060), blood urea nitrogen (*P* = 0.179), uric acid (*P* = 0.176), direct bilirubin (*P* = 0.086), serum sodium (*P* = 0.402), or serum potassium (*P* = 0.100) between groups. Neither the SOFA score nor ICU stay time at admission was significantly different. However, the SOFA scores at both day 3 and day 7 were significantly lower in the non-survivor group than in the survivor group (both *P* ≤ 0.001). The comorbidity rates of hypertension (*P* = 0.426), diabetic mellitus (*P* = 0.113), infection (*P* = 0.289), chronic renal failure (*P* = 0.895), and respiratory disease (*P* = 0.579) were also not different between the two groups. We also evaluated the organ dysfunction of patients after admission, and found no significant differences in kidney, liver, heart, and respiratory dysfunction between groups (*P* > 0.05). However, the septic shock occurrence rate was higher in the non-survivor group (*P* < 0.001).

### Changes of PLS at early stage

To investigate the difference of PLS between survivors and non-survivors, we compared the number changes of PLS between groups at early stage. In both groups, the counts of lymphocytes, CD3^+^ T cells, CD4^+^ T cells, CD8^+^ T cells, CD19^+^ B cells, and NK cells significantly decreased between day 1 and day 3 (Table 2). There were no significant differences in the counts of lymphocytes (*P* = 0.912), CD3+ T cells (*P* = 0.491), CD4+ T cells (*P* = 0.149), CD8+ T cells (*P* = 0.531), CD19+ B cells (*P* = 0.903), or NK cells (*P* = 0.633) at day1 between the two groups. However, at day 3, the counts of lymphocytes, CD3+ T cells (*P* < 0.001), CD4+ T cells (*P* < 0.001), and NK cells (*P* = 0.013) were remarkedly lower in the non-survivor group (Figure 1). No significant differences in CD8^+^ T cells (*P* = 0.403) or CD19^+^ B cells (*P* = 0.691) were found.

**Table 2:** Comparisons of early-stage changes of peripheral lymphocyte subsets between survivor group and non-survivor group (median and quartile).

**Parameters**	**Stage**	**Survivor group**	**Non-survivor group**	***P***
Lymphocytes	Day 1	798.1 (369.4–1192.8)	799.9 (524.2–1083.9)	0.912
	Day 3	353.8 (166.1–566.2)^*^	287.9 (190.1–400.2)^*^	**<0.001**
CD3^+^ T cells	Day 1	423.1 (391.2–467.0)	422.5 (392.7–451.4)	0.491
	Day 3	341.4 (318.2–361.6)^*^	321.2 (309.1–334.4)^*^	**<0.001**
CD4^+^ T cells	Day 1	539.6 (218.5–783.8)	527.7 (287.5–751.0)	0.149
	Day 3	352.8 (1117.3–624.4)	298.1 (261.9–333.6)^*^	**<0.001**
CD8^+^ T cells	Day 1	271.9 (155.8–403.2)^*^	269.2 (166.8–348.9)	0.531
	Day 3	222.3 (173.5–282.7)	220.5 (194.3–244.8)^*^	0.403
CD19^+^ B cells	Day 1	218.2 (95.5–333.9)	213.4 (155.8–320.9)	0.903
	Day 3	119.3 (34.1–226.0)^*^	124.3 (103–150.1)^*^	0.691
NK cells	Day 1	311.3 (178.6–432.6)	309.6 (231.2–388.9)	0.633
	Day 3	150.1 (88.8–211.6)^*^	121.4 (92.4–150.7)^*^	**0.013**

^*^The numbers of lymphocyte subsets are lower at day 3 than day 1.

### PLS index

For PLS, we performed multivariate logistic regression using the enter method, and obtained the regression coefficient with significant level. Results showed the numbers of lymphocytes, CD3+ T cells, CD4+ T cells, and NK cells were significantly associated with the mortality of sepsis. Then we calculated the PLS index as follows: PLS index = −0.005 × lymphocyte −0.200 × CD3-0.006 × CD4-0.036 × NK3.

### Establishment of mortality risk prediction model in sepsis

We further evaluated the association between PLS and sepsis prognosis. The 28-day mortality was considered as a dependent variable. We first performed univariate logistic regression (Supplementary Table 1). The PLS index was significantly associated with the 28-day mortality in sepsis. We also found the levels of Hb (*P* = 0.022), Plt (*P* = 0.017), lymphocytes (*P* < 0.001), CD3+ T cells (*P* < 0.001), CD4+ T cells (*P* < 0.001), and NK cells (*P* < 0.001) were negatively associated with the 28-day mortality of sepsis. The RDW (*P* < 0.001), neutrophil (*P* = 0.002), FBG (*P* = 0.001), CR (*P* = 0.026), TB (*P* = 0.006), ALB (*P* = 0.021), lactate (*P* = 0.009), serum P (*P* < 0.001), SOFA at day 3 (*P* = 0.001), SOFA at day 7 (*P* < 0.001), kidney (*P* = 0.020) and liver dysfunction (*P* = 0.007) were all positively associated with the 28-day mortality of sepsis. Other parameters were not associated with sepsis progression.

**Figure 2:**
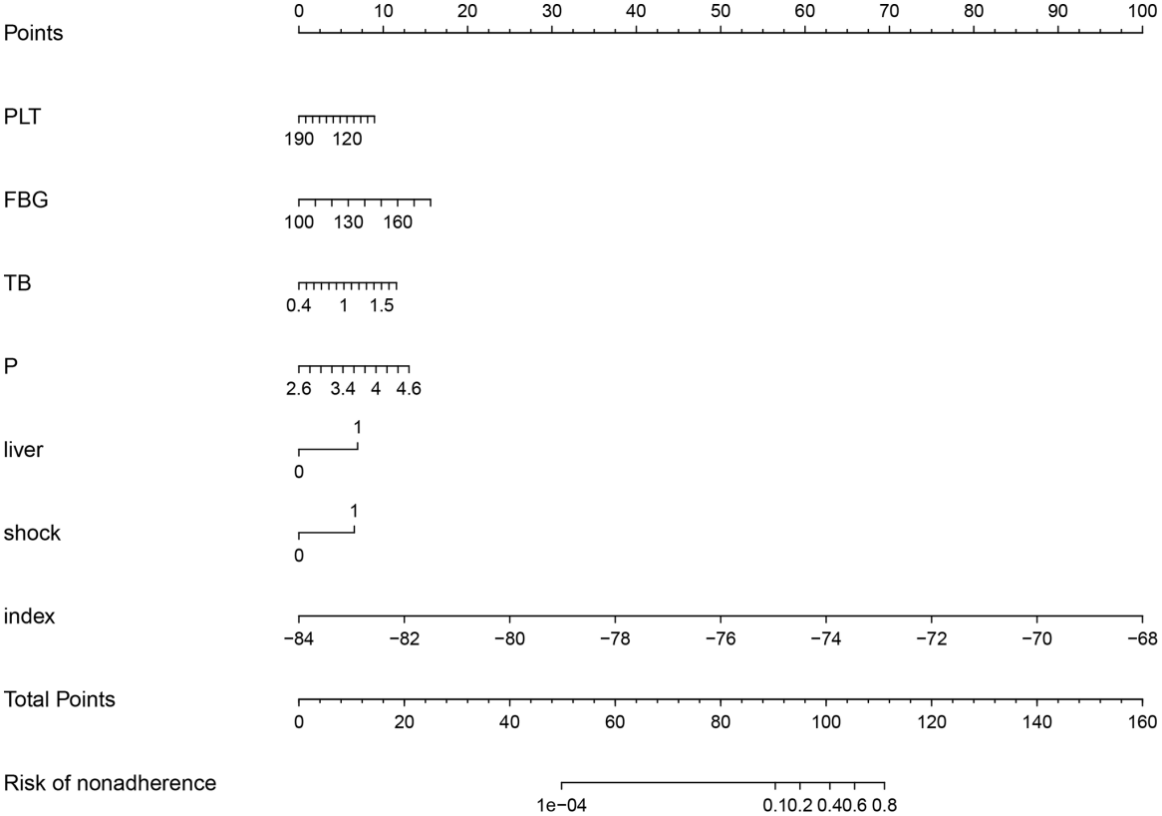
Nomograph of 28-day mortality risk prediction based on clinical parameters and the peripheral lymphocyte subset index.

Then we performed multivariate logistic regression including the significant variables in the univariate logistic regression. Results demonstrate a larger PLS index will increase the mortality risk in sepsis (OR (95% CI): 3.08 (1.18−9.67), *P* < 0.001). Besides, the Plt (0.99 (0.97−1.00), *P* = 0.041), FBG (1.04 (1.02−1.06), *P* = 0.001), TB (5.40 (1.56−18.73), *P* = 0.008), serum *P* (3.82 (1.71−8.53), *P* = 0.001), liver (3.38 (1.18−9.67), *P* = 0.001) and shock (3.37 (1.51−7.53), *P* = 0.003) were significantly associated with the 28-day mortality of sepsis (Table 3). Based on these clinical parameters and the PLS index, we developed a nomograph for evaluating the individual mortality risk in sepsis (Figure 2).

**Table 3:** Multivariate logistic regression for 28-day mortality in patients with sepsis.

**Variable**	**Beta**	**SE**	**Wald**	***P***	**OR**	**95% CI**	
Platelets	−0.015	0.007	4.175	**0.041**	0.99	0.97	1.00
Blood glucose	0.038	0.011	10.997	**0.001**	1.04	1.02	1.06
Creatinine	0.011	0.006	3.788	0.052	1.01	1.00	1.02
Total bilirubin	1.686	0.635	7.055	**0.008**	5.40	1.56	18.73
Albumin	1.160	0.619	3.513	0.061	3.19	0.95	10.73
Lactate	0.831	0.498	2.783	0.095	2.29	0.86	6.09
Serum phosphorus	1.341	0.410	10.715	**0.001**	3.82	1.71	8.53
PLS index	1.124	0.136	68.325	**0.000**	3.08	2.36	4.02
Liver dysfunction	1.218	0.536	5.160	**0.023**	3.38	1.18	9.67
Shock	1.216	0.410	8.790	**0.003**	3.37	1.51	7.53

### Assessment of mortality risk prediction models in sepsis

**Figure 3:**
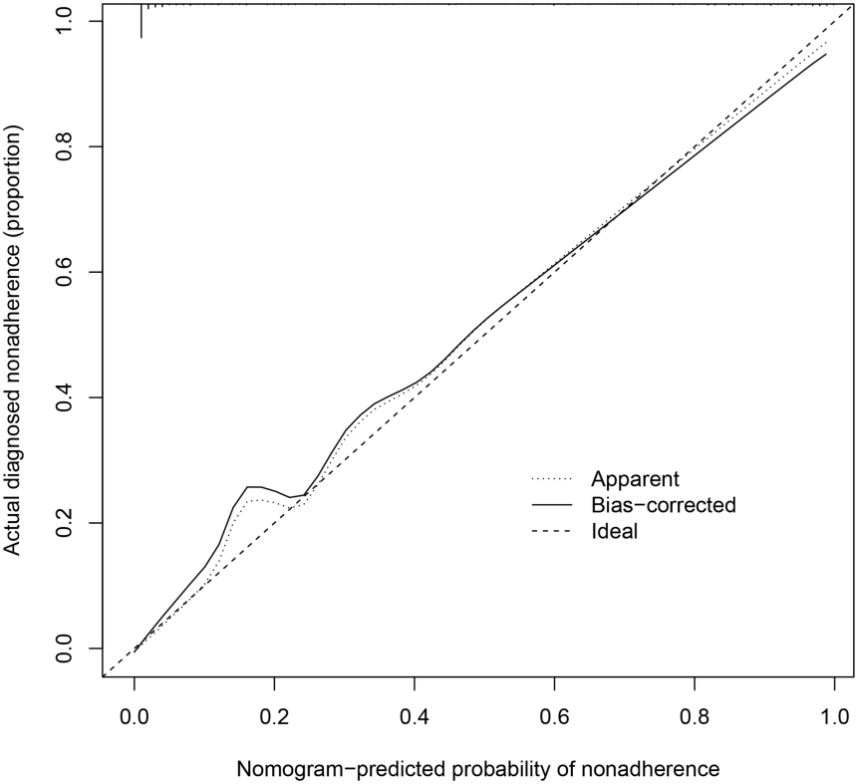
Calibration curves of 28-day mortality risk prediction in septic patients.

We further evaluated the prediction ability of each model based on clinical parameters and the PLS index. Calibration curves were used to assess the relationship between nomogram-predicted probability of nonadherence and actual diagnosed nonadherence. Figure 3 showed the predicted probability basically fitted with the actual diagnosed probability.

**Figure 4:**
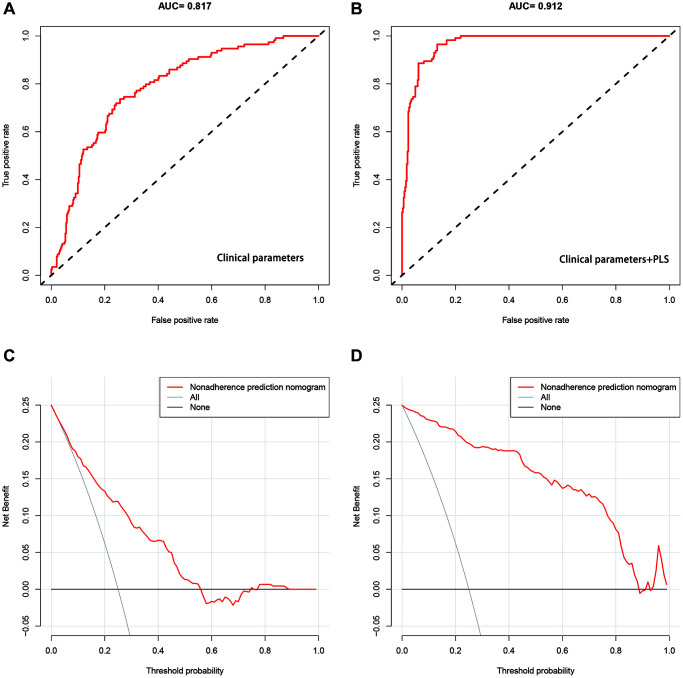
**Assessment of 28-day mortality risk prediction models in septic patients.** (**A**, **B**) ROCs of the model only including clinical parameters, and the model including clinical parameters and the PLS index respectively. (**C**, **D**) Decision curves for the model with only clinical parameters, and the model with clinical parameters and the PLS index respectively.

Then we estimated the ROC of prediction with or without the PLS index. Results show the AUC of prediction with the PLS index is 0.912 (95% CI: 0.857–0.964), which is significantly higher than the AUC (0.817, 95% CI: 0.754–0.889) from the model with only clinical parameters (Figure 4A, 4B). Furthermore, decision curve analysis found the model with the PLS index outperformed the model with only clinical parameters when the threshold probability was not less than 0.3 (Figure 4C, 4D). Patients benefited from the nonadherence prediction nomogram when the threshold was between 0.3 to 0.9.

## DISCUSSION

Our study had several findings. (1) PLS changed at early stage of sepsis. (2) The PLS index based on lymphocyte, CD3^+^ T cells, CD4^+^ T cells and NK cells was associated with the 28-day mortality in septic patients. (3) A 28-day mortality prediction model for septic patients was established using clinical parameters and the PLS index. This model has high prediction ability and brings benefits for risk management of septic patients. Our study provides new insights for evaluating the prognosis of septic patients and carrying out risk management.

Sepsis is a high-risk factor for non-survivors in severe patients [16]. A study reviewing the mortality of septic patients in Europe, North America, and Australia from 2009 to 2019 found that the average 30-day mortality rate of sepsis was 24.4% [17], which is consistent with our results that the 28-day mortality rate of septic patients is 25.0%. Early and accurate identification of septic patients at high risk of in-hospital death is helpful for ICU physicians to make the best clinical decision, thus improving clinical efficacy [18]. Reportedly, factors such as 24-hour mean serum lactate level and mean arterial pressure are independently correlated with ICU and hospital mortality [19]. Moreover, the use of vasopressors, the use of ventilators, urine volume, RBC distribution width, ICU type, malignant tumor, and metastatic solid tumor have significant predictive effects on 30-day death in elderly septic patients [20]. Additionally, FBG, CR, TB, ALB, lactate, serum phosphorus, liver dysfunction and shock are risk factors for death of septic patients.

Impaired lymphocyte function and loss of immune function are among the immunosuppressive factors in septic patients [21]. Reportedly, the proportions of T lymphocyte subsets and dysfunction significantly decrease in septic patients. Furthermore, T lymphocytes not only clear target cells through specific killing, but also transmit signals by responding to antigens and assist B lymphocytes to participate in maintaining the homeostasis of the humoral immune system [22]. When sepsis occurs, both pro-inflammatory and anti-inflammatory processes occur in the host immunity, and the balance between them determines the pathological progress and clinical outcome [23]. Previously, an uncontrolled and amplifying pro-inflammatory response was initially believed to be the major cause of mortality in sepsis [24]. However, recent studies show that validation response and immunosuppression may occur simultaneously during sepsis, implying that the early immune status of septic patients has been altered [25]. As helper T cells, CD4+T lymphocytes play an important role in the immune system of the body and maintain system stability. When pathogens invade the body, CD4+T lymphocytes are activated and can differentiate into different effector lymphocytes and produce corresponding cytokines according to their different functions, including cellular immunity involving T helper (Th) 1 [26]. Th1 can secrete interleukin (IL)-2 and tumor necrosis factor (TNF)-α. Th2 cells mediate humoral immunity, and secret IL-3, IL-4, and IL-13. Regulatory T lymphocytes (Tregs) maintain the stability of the immune state of the body through correlation and interaction [27]. In addition, phenotypic changes of CD8+T cells in septic patients can reduce the efficacy of CD8+T cells in fighting infection [28], further leading to immunosuppression. Like CD4+T cells, the increased apoptosis of CD8+T cells during sepsis is another main mechanism of immunosuppression in sepsis, and is correlated with mortality. Yang et al. found that the absolute values of lymphocytes, CD3+, CD3+CD4+, and CD19+ in non-survivors were lower than those in survivors [29]. Tang et al. reported the CD8+ T cell count was predictive of sepsis progression. Depletion of lymphopenia and CD8+ T cells was associated with the clinical outcomes of sepsis, suggesting that CD8+ T cells are a potential predictive biomarker and therapeutic target for septic patients [30]. Our results also showed the counts of lymphocytes, CD3+ T cells, CD4+ T cells, and NK cells on day 3 declined significantly in the dying group compared to the surviving group. This result also proves that early immunosuppression of sepsis has begun. As reported, the absolute counts of CD3+ T cells, CD4+ T cells, CD8+ T cells, B cells, and natural killer cells were associated with clinical prognosis in septic patients. Especially, the CD8+ T cell count was predictive of sepsis progression, and lymphopenia and CD8+ T cell depletion was associated with the clinical outcomes of sepsis [30–32]. Here, we did not analyze the association between each lymphocyte subset and clinical outcomes. We constructed a PLS index based on lymphocyte subsets, including lymphocytes, CD3, CD4, and NK cells. Multivariate logistic regression revealed that the PLS index was an independent risk factor for 28-day death in septic patients.

The current research on sepsis-induced death prediction can be broadly divided into two categories: regression-based scoring systems and machine learning-based predictive models. Several scoring systems have been used to assess the severity of illness and risk of death in critically ill patients. APACHE-II, SAPS II, SOFA, and MODS scores are mostly used. Due to large clinical heterogeneity, the prediction of death risk in septic patients by these systems is not reliable [33–35]. To better predict sepsis death, many machine learning models have been applied to establish prognostic models of sepsis death and development [36, 37]. In some research, the MIME-III database was used to build a tool for predicting the risk of death in septic patients, which indicates the machine learning prediction models superior over the SOFA score in discrimination [38]. Zhi et al. established an in-hospital death prediction model in septic patients based on MIME-iii database using random forest method and logistic regression, and found that the random forest model had good discriminant ability, especially in the population with SOFA value of 13–15 [39]. Rodriguez et al. used four machine learning methods to establish a model for predicting in-hospital death of septic patients, and reported that support vector machine (SVM) and artificial neural network (ANN) were the best performing models, with an AUC of 0.690 [40]. Li et al. compared five machine learning models in predicting hospitalization death, and found that the gradient lifting decision tree model performed the best, with an AUC of 0.992 [41]. The gradient lifting machine model with an AUC of 0.845 outperformed LASSO, random forest, logistic regression, and SAPS II models in predicting in-hospital death [42]. In conclusion, the AUC of the existing sepsis death prediction models ranges from 0.690 to 0.992, but there is a lack of model prediction studies based on lymphocyte subsets. We constructed a 28-day death prediction model for sepsis based on clinical parameters and the PLS index. In comparison with the model containing only clinical parameters (AUC: 0.817), and the predictive power of the model was greatly improved after adding PLS index (AUC: 0.912), which had relatively high ability in predicting the 28-day mortality of septic patients.

Our study has several limitations. First, this study focused on changes in the levels of lymphocyte subsets at the early stage of sepsis, which was a shorter time span compared to the length of hospitalization of septic patients. Second, we did not detect other immunological indicators, inflammatory mediators, or lymphocyte functions, such as antigen presenting cells, human leukocyte DR antigen, interleukin, tumor necrosis factor, and C-reactive protein, so this study may not be accurate in judging the immune status of the body. In addition, this study only covered a survival group and a non-survivor group divided according to the outcome of septic patients, but did not involve ICU patients with non-sepsis or healthy volunteers. Finally, as this is a single-center study with a small sample size, multicenter studies are needed to further evaluate the value of lymphocyte subset changes predicting the 28-day prognosis of septic patients.

In conclusion, the decline of PLS occurred in the early stage of sepsis. A new novel PLS index can be used as an independent predictor of 28-day mortality in septic patients. The prediction model based on clinical parameters and the PLS index has higher predicting ability. Our study provides new insights for evaluating the prognosis of septic patients and carrying out risk management in clinical practice.

## Supplementary Materials

Supplementary Table 1
