# Supplementary material for: Establishment and assessment of mortality risk prediction model in patients with sepsis based on early-stage peripheral lymphocyte subsets
Source: Aging (Albany NY). 2024 Apr 25;16(8):7460–73. doi: 10.18632/aging.205772 (PMC11087126; doi:10.18632/aging.205772)
Supplement: Supplementary Table 1 [file aging-16-205772-s001.pdf]

SUPPLEMENTARY TABLE

Supplementary Table 1. Univariate logistic regression for 28-day mortality in patients with sepsis.

Variable	Beta	SE	Wald	*P*	OR	95% CI	
Sex	0.346	0.230	2.262	0.133	1.41	0.90	2.22
Age	0.001	0.006	0.065	0.800	1.00	0.99	1.01
Body mass index	0.019	0.017	1.343	0.247	1.02	0.99	1.05
Pulmonary	0.047	0.217	0.047	0.828	1.05	0.69	1.60
Cardiovascular	0.346	0.232	2.221	0.136	1.41	0.90	2.23
Infectious diseases	−0.300	0.264	1.288	0.257	0.74	0.44	1.24
Polytrauma	0.463	0.319	2.101	0.147	1.59	0.85	2.97
Gastrointestinal bleeding	0.127	0.407	0.098	0.754	1.14	0.51	2.52
White blood cells	−0.060	0.036	2.666	0.103	0.94	0.88	1.01
Red blood cells	−0.153	0.082	3.507	0.061	0.86	0.73	1.01
Hemoglobin	−0.397	0.173	5.276	**0.022**	0.67	0.48	0.94
Red cell distribution width	0.680	0.120	32.008	**<0.001**	1.97	1.56	2.50
Platelets	−0.009	0.004	5.712	**0.017**	0.99	0.98	1.00
Neutrophil	0.060	0.020	9.188	**0.002**	1.06	1.02	1.10
Blood glucose	0.018	0.006	10.249	**0.001**	1.02	1.01	1.03
Blood urea nitrogen	0.020	0.015	1.807	0.179	1.02	0.99	1.05
Creatinine	0.007	0.003	4.937	**0.026**	1.01	1.00	1.01
Uric acid	0.002	0.002	1.832	0.176	1.00	1.00	1.01
Total bilirubin	0.899	0.329	7.476	**0.006**	2.46	1.29	4.68
Direct bilirubin	0.331	0.193	2.945	0.086	1.39	0.95	2.03
Albumin	0.798	0.347	5.297	**0.021**	2.22	1.13	4.38
Lactate	0.733	0.278	6.929	**0.009**	2.08	1.21	3.59
Serum sodium	0.023	0.028	0.706	0.401	1.02	0.97	1.08
Serum potassium	0.541	0.329	2.704	**0.100**	1.72	0.90	3.27
Serum phosphorus	0.955	0.223	18.359	**<0.001**	2.60	1.68	4.02
SOFA	−0.006	0.059	0.010	0.920	0.99	0.89	1.12
SOFA3	0.233	0.071	10.705	0.001	1.26	1.10	1.45
SOFA7	0.267	0.062	18.757	<0.001	1.31	1.16	1.47
Hypertension	0.184	0.231	0.632	0.427	1.20	0.76	1.89
Diabetic mellitus	0.351	0.223	2.493	0.114	1.42	0.92	2.20
Infection	0.288	0.272	1.121	0.290	1.33	0.78	2.27
Chronic renal failure	0.035	0.262	0.018	0.895	1.04	0.62	1.73
Respiratory disease	0.124	0.223	0.307	0.580	1.13	0.73	1.75
**Organ dysfunction**							
Kidney	0.506	0.218	5.412	**0.020**	1.66	1.08	2.54
Liver	0.743	0.273	7.395	**0.007**	2.10	1.23	3.59
Heart	0.131	0.219	0.358	0.550	1.14	0.74	1.75
Lung	0.127	0.362	0.124	0.725	1.14	0.56	2.31
Shock	1.570	0.230	46.444	**<0.001**	4.80	3.06	7.55
**Lymphocyte subsets at day 1**							
Lymphocytes	0.000	0.001	0.041	0.841	1.00	1.00	1.00
$CD3^+$ T cells	−0.009	0.008	1.090	0.297	0.99	0.98	1.01
$CD4^+$ T cells	−0.002	0.001	1.867	0.172	1.00	1.00	1.00
$CD8^+$ T cells	−0.002	0.002	0.417	0.518	1.00	0.99	1.00

CD19^{+} T cells	0.002	0.003	0.275	0.600	1.00	1.00	1.01
NK cells	−0.001	0.003	0.109	0.741	1.00	0.99	1.00
**Lymphocyte subsets at day 3**							
Lymphocytes	−0.006	0.001	25.523	**<0.001**	0.99	0.99	1.00
CD3^{+} T cells	−0.186	0.020	89.150	**<0.001**	0.83	0.80	0.86
CD4^{+} T cells	−0.004	0.001	18.456	**<0.001**	1.00	0.99	1.00
CD8^{+} T cells	−0.006	0.004	2.266	0.132	0.99	0.99	1.00
CD19^{+} B cells	−0.001	0.002	0.155	0.694	1.00	1.00	1.00
NK cells	−0.032	0.004	53.369	**<0.001**	0.97	0.96	0.98
